# An improved pear disease classification approach using cycle generative adversarial network

**DOI:** 10.1038/s41598-024-57143-6

**Published:** 2024-03-20

**Authors:** Khulud Alshammari, Reem Alshammari, Alanoud Alshammari, Tahani Alkhudaydi

**Affiliations:** Present Address: Faculty of Computers & Information Technology, University of Tabuk, Tabuk, Saudi Arabia

**Keywords:** Deep vision classification approaches, CycleGAN, Plant disease classification, DiaMOS dataset, Evolution, Environmental sciences

## Abstract

A large number of countries worldwide depend on the agriculture, as agriculture can assist in reducing poverty, raising the country’s income, and improving the food security. However, the plan diseases usually affect food crops and hence play a significant role in the annual yield and economic losses in the agricultural sector. In general, plant diseases have historically been identified by humans using their eyes, where this approach is often inexact, time-consuming, and exhausting. Recently, the employment of machine learning and deep learning approaches have significantly improved the classification and recognition accuracy for several applications. Despite the CNN models offer high accuracy for plant disease detection and classification, however, the limited available data for training the CNN model affects seriously the classification accuracy. Therefore, in this paper, we designed a Cycle Generative Adversarial Network (CycleGAN) to overcome the limitations of over-fitting and the limited size of the available datasets. In addition, we developed an efficient plant disease classification approach, where we adopt the CycleGAN architecture in order to enhance the classification accuracy. The obtained results showed an average enhancement of 7% in the classification accuracy.

## Introduction

Recently, the adoption of machine learning methods has attained high performance in many computer vision tasks^1–3^. However, machine learning methods employ statistical learning algorithms to explore patterns in the training subset and then perform predictions and classification on the test subset, whereas deep neural networks choose features, provide an end-to-end pipeline for automatically extracting robust features, significantly improving the availability of leaf identification.

On the other hand, the identification of leaf diseases is essential for controlling disease spread and improving the healthy development of the pear industry^4–6^. However, plant disease prediction is a hard and interconnected task that needs different technical skills and experts in the field. The existing traditional approaches depend on a specialist or expert in the field to manually perform careful analysis of the foliar surface and then perform a diagnosis, but these approaches are inefficient in terms of complexity and cost^7,8^. Therefore, the deployment of technology has become necessary for automatically recognizing and classifying the plant diseases from the appearance of the first symptoms.

Machine learning and deep learning approaches have received considerable attention recently for the purpose of object identification and classification^9^. Recently, there are several plant leaf diseases approaches that have been proposed^10–12^, which are based on the employment of deep neural networks. These approaches showed that deep learning approaches have enhanced the classification accuracy in a comparison with the machine learning approaches. This is due to the large number of configurable parameters in the Deep Convolutional Neural Network (DCNN), where a substantial amount of labelled data is needed to train the model and improve the generalization capabilities.

The CNN models require enough number of training images in order to enhance the generalization capabilities. However, there is a lack of agricultural data, particularly in the field of identifying leaf diseases, as the collection process of huge disease datasets is inefficient in terms of labor and time, since this type of data requires an extensive knowledge in the area of plant diseases. In addition, the manual labelling task is a very subjective task, and ensuring the quality of the classified data is difficult^13^.

As a result, the lack of training samples is the primary constraint to further improvement for the accuracy of leaf disease detection. Hence, the problem of train a deep learning model using a small amount of labelled data is worth examining. This problem has been addressed using the employment of traditional data augmentation methods^14^.

Recently, the adoption of data augmentation approaches in computer vision tasks enhances the quality of deep learning classification, through obtaining a large size dataset, and hence better deep learning models can be trained with the improved datasets^15^. Therefore, since image data is distinct, a new training dataset can be extracted from the original image using a simple geometric transformation, including: rotation, scaling, translation, cropping, noise addition, and other data augmentation techniques. These strategies, however, provide very extra information, and hence improving the accuracy for classification objects in a certain area. Several approaches have considered the issue of data augmentation to enhance the classification accuracy, as presented in^10,16–19^, where the most popular approach for training a deep CNN model with a small amount of data is to augment the input data with synthetic images.

In this research work, we designed the Generative Adversarial Network (GAN) to obtain new synthetic images that are augmented the training dataset. GAN is a machine learning-based approach that is utilized to produce additional new samples with similar features to the original samples, in order to be employed in the training process. GAN aims to generate entire synthetic images that can contribute to increase the dataset’s diversification. Recently, GAN approaches have become a standard method for dealing with dataset constraints. On the other hand, to mitigate the bias induced by class imbalance, authors of^17^ proposed a new approach to augment synthetic samples named as Activation Reconstruction (AR).

Unlike the work presented in the previous research works^10,16,17,20^ which consists of 9 different classes with balanced dataset, our research work focuses on the same issue with imbalanced dataset. Therefore, our main target is to increase the training samples and balance the number of records in each class, in order to minimize the model over-fitting, and hence offers high classification accuracy.

Therefore, in this work, we develop an efficient deep vision classification approach for pear plant disease classification through the employment the Cycle GAN, in order to increase the number of samples in the selected dataset through producing new images, that result in enhancing the classification accuracy. Hence, the main contribution of this work lies on the following aspects:

1. Develop a new efficient approach that integrates classification with the CycleGAN approach in order to resolve the problem of a limited training set.
2. Validate the proposed approach through several experiments to assess the performance of the developed system in terms of classification accuracy.

The rest of this paper is organized as follows: “Related works” section discusses the recent developed pear plant disease classification approaches, whereas “System design” section presents and discusses the proposed classification system. “Experimental results” section shows the experimental testbeds and results, and “Discussion” section discusses the results obtained from several experiments that have been conducted to assess the efficiency of the developed system. And finally, “Conclusion and future work” section concludes the work presented in this paper and draws a future work.

## Related works

This section discusses the recent developed plant disease classification approaches detection. Authors of^21^ reviews the diseases affecting on 11 different plants and presented how the diseases can be identified from plant leaf images using deep CNN learning models. However, in this paper, we focus on the problem of classification of pear diseases through the employment of deep CNN models.

The work presented in^22^ involves exploring the variability factors that impact the classification of plant diseases through analyzing the same plant and diseases under different conditions, where 5 different approaches including EfficientB0, MobileNetV2, InceptionV2, ResNet50, and VGG16 have been employed. As a result of this study, authors revealed that the model performance drops extremely when using representative datasets, in addition, the presented results showed that deep learning is an effective technique for plant disease classification.

Authors of^23^ investigated the Convolutional Block Attention Module (CBAM) to enhance the classification accuracy of CNN which is a lightweight attention module which can be plugged into the CNN architecture with negligible overhead. Authors employed several CNN models, including: EfficientNetB0, MobileNetV2, ResNet50, InceptionV3, and VGG19 in order to perform transfer learning for plant disease classification. As a result, the EfficientnetB0 and CBAM architecture obtained 86.89% classification accuracy.

In^11^, authors investigated the ensemble learning approach to develop a robust network that is able to predict four different pear leaf diseases, where authors employed and analyzed the results of several CNN architectures, including: EfficientNetB0, InceptionV3, MobileNetV2 and VGG19.

The work presented in^24^ involves employing an improved YOLOv4-tiny for the purpose of root crop detection using a single-board computer (SBC). The employed method allows for processing up to 14 images of 416 × 416 pixels with a result of 86% precision and 91% recall.

The task of balancing the dataset in deep learning applications is a crucial problem that has been researched and addressed in several research works, driven by the requirement of a health agricultural yield, whereas cost-effectiveness, accuracy, sensitivity, and user-friendliness are all essential considerations.

Although data augmentation is an effective strategy to deal with limited training data, however it is not widely applicable. Because selecting a suitable data augmentation technique requires a prior understanding of the target domain and task. Furthermore, parametrization of data augmentation methods adds a new set of key hyper-parameters that can significantly affect the deep learning method's inaccuracy.

Therefore, in order to balance skewed data, authors of^19^ proposed a data augmentation approach through employing Deep Convolutional Generative Adversarial Networks (DCGAN). Authors revealed that the best classification accuracy is achieved when the number of samples in each class is nearly equal (balanced dataset). Authors employed a VGG16 CNN model as a classifier to assess the performance of the developed DCGAN approach. The experimental results revealed that, with employing the GAN-based data augmentation strategies, an increase in the distribution integrity of classes in the selected dataset.

As presented above, various GAN methods have been employed in diverse deep neural network application in order to address the challenge of balancing imbalanced datasets. DCGAN on the other hand employs random vector image synthesis rather than image translation, which can result in a bottleneck. However, with unpaired dataset, DCGAN method does not consider image-to-image translation.

In addition, large amount of data is necessary for the purpose of improvement of the classification accuracy in all of the above approaches, as well as other deep learning approaches for various tasks in computer vision so far. Starting with a small number of original images and gradually synthesizing additional plant disease images synthetically using CycleGAN and feeding those samples to our training set using the approach proposed in this study, we may achieve comparable performance.

## System design

In order to obtain an efficient classification performance in terms of classification accuracy for the purpose of the pear plant diseases categorization using a small number of images in the training dataset. This section discusses the system design including the design of two different approaches in order to assess the efficiency of employing the CycleGAN architecture on the classification accuracy.

For the purpose of plant disease classification, we discuss various deep CNN models which may be employed for the purpose of plant disease classification. Recently, there are several deep CNN models that have been developed for the purpose of image classification. AlexNet model^25^ has a total number of 8-layer structure with 60 million parameters, whereas VGGNet-16^26^ consists of a total number of 16 layers and 7 million parameters. GoogleNet^27^ on the other hand consists of nine inception modules and two max-pooling layers for down-sampling the input as its fed forward through the network. The Inception V3, MoibleNetV1 and MobileNetV2 consist of 27, 4.2, and 3.37 million of parameters, respectively. Table 1 summarizes the deep CNN models that have been employed for image classification tasks.

**Table 1:** Summary of the deep CNN models that have been employed for image classification.

Model	No. of layers	Parameters (million)	Size
AlexNet	8	60	–
VGGNet-16	23	138	528 MB
VGGNet-19	26	143	549 MB
Inception-V1	27	7	–
Inception-V3	42	127	93 MB
ResNet-152	152	50	132 MB
ResNet-101	101	44	171 MB
InceptionResNet V2	572	55	215 MB
EfficientNet B0	–	5	–
MobileNet-V1	28	4.2	16 MB
MobileNet-V2	28	3.37	14 MB

For the purpose of improving the classification accuracy, and according to several research works^28–30^ that analyzed the performance of several deep vision models, we have employed 3 different deep vision models: VGG19, ResNet50 and EfficientNetB0.

The developed system has been implemented through two different approaches: the first approach involves developing a plant disease classification using the original DiaMOS dataset, whereas in the second approach, we employed the CycleGAN method to efficiently increase the size of the DiaMOS dataset.

**Figure 1:**
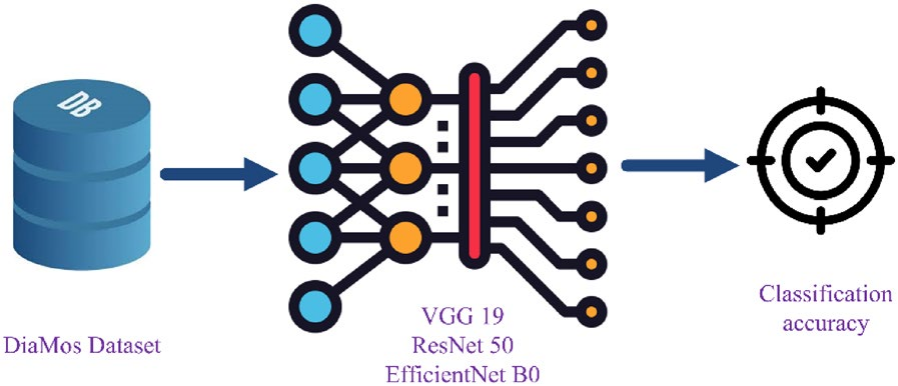
The main stages of approach 1.

The first approach is depicted in Fig. 1, where the DiaMOS dataset is first divided into two subsets: training and testing, then train the deep CNN model using 3 different deep classification models (VGG19, ResNet50 and EfficientNetB0) through employing the original DiaMOS dataset.

**Figure 2:**
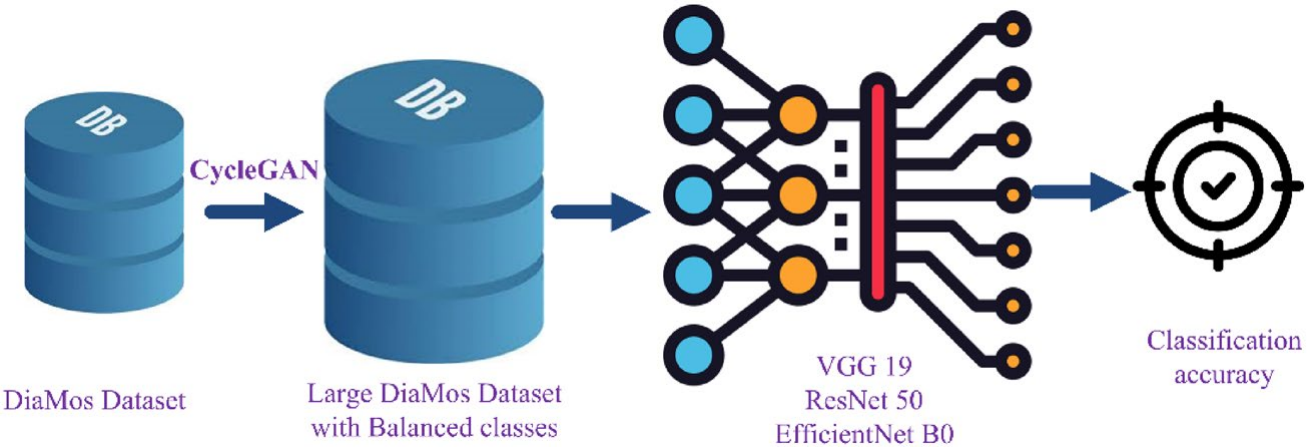
The main stages of approach 2.

The next approach involves generating new labelled images through training the CycleGAN, for two main reasons: balancing the classes in the DiaMOS dataset, and increase the size of the DiaMOS dataset in order to obtain better classification accuracy, where the new generated images will be added to the original image dataset in order to enhance the initial dataset, through incorporating more images. Next, the designed CNN model is employed to train using the new generated dataset. Figure 2 presents the concept of the second approach, where the integration of CycleGAN method is presented before the process of dividing the dataset into two subsets.

In general, the CycleGAN architecture consists of two models: the generator model and a discriminator model. The generator receives a point from a latent space as an input and produces new plausible images from the domain, whereas the discriminator takes an image as an input and predicts whether the taken image is a real one (from the DiaMOS dataset) or fake (from the generated dataset).

On the other hand, transfer learning is the reuse of a previously trained network to a new task in deep learning. Transfer learning has become popular recently as it allows the network to be trained with a minimal quantity of data while retaining high accuracy. A computer leverages information from a prior task to boost generalization about the new task in transfer learning. The final levels of the trained network are replaced in our approach by adding extra layers for the aim of transfer learning, such as adding a fully connected layer and a four-class soft-max classification layer. Therefore, we added an additional activation layer, batch-normalization layer, and a dropout layer. For the experiments, we use the same settings in terms of batch-size and learning rates.

## Experimental results

In this section, we discuss the materials and methods that been employed to develop an efficient pear plant disease classification approach. In addition, we present and discuss the results obtained from two different approaches. Therefore, for the purpose of system validation, the first stage includes the employment of the three different deep vision models with the DiaMOS dataset, and then train each deep vision model separately, whereas in the next stage, new labelled images are generated using the CycleGAN approach, where the new generated images will be added to the original dataset to build a new dataset with more images and balanced classes.

The final stage includes employing the deep vision models with the new generated dataset. Then a comparative study is provided to show the classification accuracy between the original dataset, and the new generated dataset through applying the CyleGAN approach.

### DiaMOS dataset

For the purpose of system validation, the DiaMOS dataset has been employed in our study. DiaMOS dataset is presented in^31^ and is a recent pear dataset, which consists of 3,505 images for pear fruit and leaves affected by three different diseases (spot, curl, slug), with the aim of saving time and resources for other researchers to allow more effort to be paid on the evaluation and comparison of classification algorithms. DiaMOS plant is a pilot dataset that contains images of an entire growing season of a pear tree, for the purpose of building a representative sample that covers the main cultural aspects of the pear plant. Table 2 summarizes the main attributes for the DiaMOS dataset.

**Table 2:** General information about the DiaMOS plant dataset.

Attribute	Value
Plant	Pear
Data source location	Sardegna, Italy
Region of Interest (ROI) captured	RGB imags
Cultivar	Leaf, fruit
Annotation	csv, YOLO
Total size	3505 images (3006 leaves images + 499 fruit images)

DiaMOS dataset has been employed in diverse plant disease classification works^7,11,22,23^, where DiaMOS dataset consists of four categories: healthy, leaf-spot, leaf-curl, and slug damage, as presented in Table 3.

**Table 3:** The distribution of classes in the DiaMOS plant dataset.

Leaf symptoms	Size
Healthy	43
Spot	884
Curl	54
Slug	2025

DiaMOS images were collected using different devices, including a smartphone (Honor 6x) and DSRL camera (Canon EOS 60D), therefore, DiaMOS dataset consists of two types of image resolutions: 2976 × 3968 and 3546 × 5184. However, the different resolutions increase the complexity of the DiaMOS dataset and represents an added value for the dataset itself.

In our study, we focus on classifying the pear images into 4 different classes: healthy, slug, curl, and spot. DiaMOS dataset has been divided into 3 main parts: training subset, validation subset, and testing subset, with the ratio of 70%, 10%, and 20% respectively. In addition, in order to preserve the balanced percent of each class, DiaMOS dataset was split using the ShuffleSplit technique presented in^19^. On the other hand, the dimension for each image in the DiaMOS dataset, was reduced to 224 × 224 × 3.

### Convolutional neural network classifier

Convolutional Neural Network (CNN) is a type of network architecture for deep learning algorithms which is specifically used for image recognition and the tasks that include the processing of pixel data. In this work, we employed an efficient CNN model to achieve the goal of plant disease recognition. The employed CNN model consists of several layers: convolutional layers, pooling layers, batch normalization layers, and fully connected layers.

In this paper, we have employed the VGG19, ResNet50, Inception-V3, MobileNet-V2 and EfficientNetB0 to diagnose the different plant diseases in the DiaMOS dataset. The reason behind employing these three models was because of their parameter numbers are smaller than other deep CNN models. In addition, these models require less computation requirements than other models.

### CycleGAN

The training process of deep learning model requires large number of records of paired examples. However, in most cases, preparing large datasets are not valid, as with the employed DiaMOS dataset. The CycleGAN is a method that involves an autonomous training of image-to-image translation models without paired examples using the GAN architecture, where these models are trained in an unsupervised approach, using a collection of images from the source and target domain that do not require to be related in any way.

**Figure 3:**
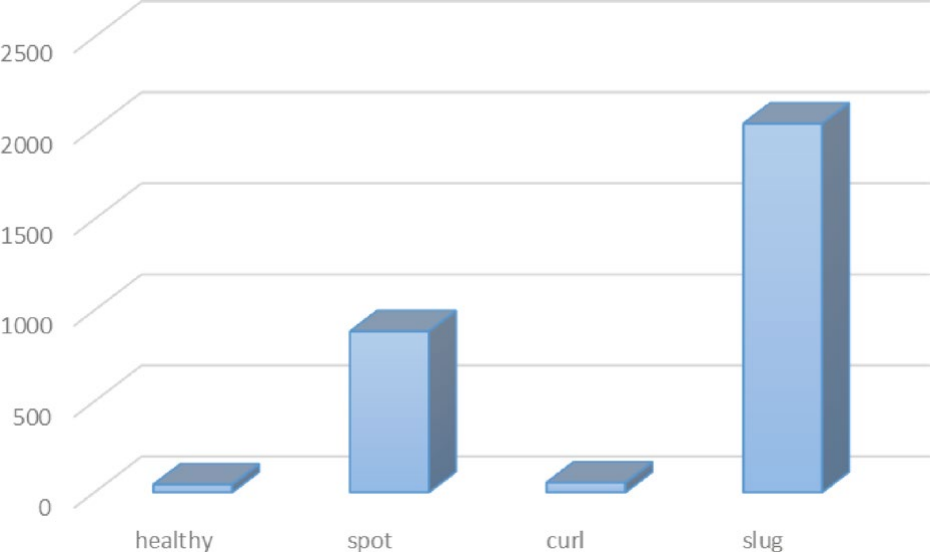
The distribution of images for each class.

As discussed earlier, DiaMOS dataset contains a small number of records, in addition, DiaMOS includes imbalanced data classes, and hence this leads to poor plant disease classification performance. Therefore, one of our main goals was to increase the size of the DiaMOS dataset and balance the records in each class. Figure 3 presents the histogram for the total number of images for each class (plant disease) located in the DiaMOS.

**Figure 4:**
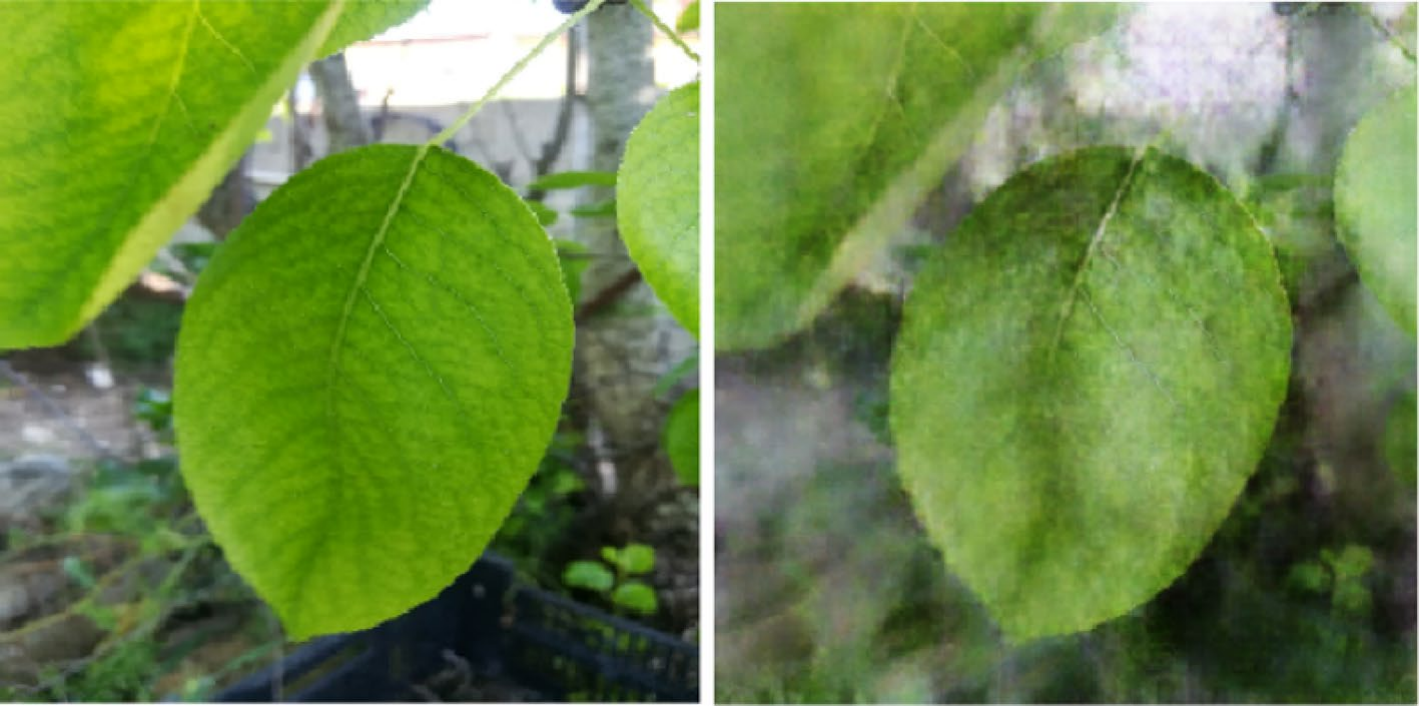
An original image of a healthy class (left-side) and a generated image by GAN (right-side).

**Figure 5:**
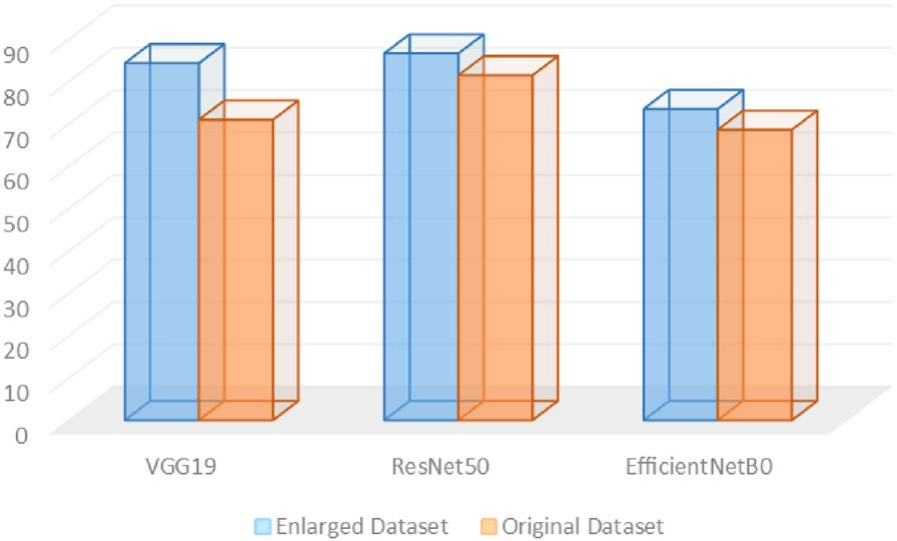
The accuracy score for the 3 models using the two datasets.

Therefore, CycleGAN has been adopted to expand the data set size, which had the same number of images. Figure 5 shows an example of a real image (left-hand side) and a sample image (right-hand side) generated by the regularized GAN architecture. Figure 4 presents an example to one unique class that belongs to the healthy class.

### Results

This section discusses the results obtained from two main different scenarios: the first one was through adopting the 3 deep vision models with the original DiaMOS dataset, whereas the second scenario involves employing the 3 deep models with the enlarged DiaMOS dataset after deploying the CycleGAN approach. For both scenarios, we estimated the following parameters:

1. Confusion matrix: in general, the confusion matrix illustrates the complete performance of the deep classification model.
2. Classification accuracy: it refers to the percentage of the total number of plant images that were correctly predicted.
3. Precision: it refers to the percentage of correctly predicted positive cases over the total predicted positive cases.
4. Recall: it refers to the percentage of correctly predicted positive cases to all the cases in the real class.
5. F1-score: it refers to the weighted average of precision and recall. In certain cases, the F1-score is more useful than classification accuracy when the dataset is unbalanced, as with the original DiaMOS dataset.

First, the results of the confusion matrix are discussed in details. Confusion matrix shows the errors that made by the classifier and the types of errors that are being made. The confusion matrices are presented first for employing the three deep vision models with the original dataset.

Table 4 presents the confusion matrix for the VGG19 model using the original dataset. As noticed, the average misclassification is equal to 29%, which is a high ratio. On the other hand, Table 5 shows the confusion matrix for the EfficientNetB0 model using the original dataset, with a higher misclassification rate of 31%. Finally, Table 6 includes the confusion matrix for the ResNet50 model with the original dataset, where a minimum misclassification rate was obtained with this model with a percentage of 18%. Therefore, the RestNet50 classification model achieves the best results in terms of classification accuracy.

**Table 4:** Confusion matrix results for the original dataset using the VGG19 model.

Actual	Healthy	0	2	2	0
	Slug	0	187	16	0
	Spot	0	66	23	0
	Curl	0	0	0	0
		Healthy	Slug	Spot	Curl
		Predicted

**Table 5:** Confusion matrix results for the original dataset using the EfficientNetB0 model.

Actual	Healthy	0	4	0	0
	Slug	0	203	0	0
	Spot	0	89	0	0
	Curl	0	0	0	0
		Healthy	Slug	Spot	Curl
		Predicted

**Table 6:** Confusion matrix results for the original dataset using the ResNet50 model.

Actual	Healthy	0	0	4	0
	Slug	0	175	28	0
	Spot	0	23	66	0
	Curl	0	0	0	0
		Healthy	Slug	Spot	Curl
		Predicted			

Then, the confusion matrices are presented for employing the three deep vision models with CycleGAN leaf images dataset. Table 7 presents the confusion matrix for the VGG19 model using the CycleGAN architecture. On the other hand, Table 8 presents the confusion matrix for the CycleGAN approaching using the ResNet50 model. As noticed, ResNet50 achieves better classification accuracy than the VGG19 model. And finally, Table 9 presents the confusion matrix for the EfficientNetB0 model using the CycleGAN leaf images.

**Table 7:** Confusion matrix results for the leaf images dataset using the VGG19 model.

Actual	Healthy	45	0	0	0
	Slug	2	363	63	0
	Spot	0	43	112	8
	Curl	0	0	0	102
		Healthy	Slug	Spot	Curl
		Predicted

**Table 8:** Confusion matrix results for the leaf images dataset using the ResNet50 model.

Actual	Healthy	32	0	0	0
	Slug	7	170	33	1
	Spot	0	10	79	4
	Curl	0	0	0	74
		Healthy	Slug	Spot	Curl
		Predicted

**Table 9:** Confusion matrix results for the leaf images dataset using the EfficientNetB0 model.

Actual	Healthy	29	0	6	4
	Slug	5	172	27	0
	Spot	0	40	51	25
	Curl	2	0	0	50
		Healthy	Slug	Spot	Curl
		Predicted			

The accuracy, precision, recall, and F1-score are significant parameters that need to be measured for each deep vision model. According to experimental studies, ResNet50 offers the best classification accuracy using the original DiaMOS dataset. In addition, ResNet50 achieves the best precision, recall, and F1-score among the three models. Table 10 summarizes the results of accuracy, precision, recall, and F1-score for the three deep vision models using the original dataset.

**Table 10:** The results obtained from 3 different models using the original DiaMOS dataset.

Architecture	Accuracy (%)	Precision (%)	Recall (%)	F1-score (%)
VGG19	70.95	17.14	25.00	20.34
ResNet50	81.42	38.93	40.09	39.48
EfficientNetB0	68.58	17.14	25.00	20.34

Table 11 on other hand presents the experimental results of accuracy, precision, recall, and F1-score for the three deep vision models using the CycleGAN leaf images. As noticed, the classification accuracy has improved for the ResNet50 model when the CycleGAN leaf images were employed, and this refers to the high efficiency of the CycleGNA architecture. Moreover, the precision score has significantly enhanced with the VGG19 and ResNet50 models. The recall parameter metric has also improved significantly with 91% for the ResNet50 deep vision model. And finally, the F1-score has also enhanced for the ResNet50 model with 87% score.

**Table 11:** The results obtained from 3 different models using the CycleGAN leaf images.

Architecture	Accuracy (%)	Precision (%)	Recall (%)	F1-score (%)
VGG19	84.28	85.47	88.38	86.84
ResNet50	86.59	85.17	91.38	87.73
EfficientNetB0	73.48	71.42	74.70	71.84

As noticed from the experimental results, the accuracy, precision, recall, and F1-score have improved for all the deep vision models when adopting the CycleGAN leaf images. Figure 5 shows the classification accuracy for both the original DiaMOS dataset and the CycleGAN leaf images dataset. As presented below, the classification accuracy has improved for the three adopted models with an average of 7% enhancement.

**Figure 6:**
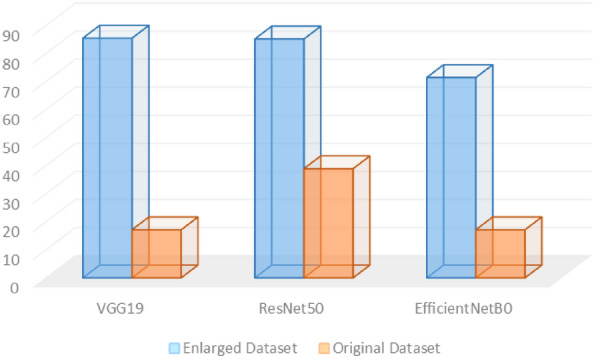
The precision score for the 3 models using the two datasets.

The precision score was measured in both scenarios for the three deep vision approaches, as noticed in Fig. 6. As presented below, there is a significant difference between the precision scores using the original dataset, and with using the CycleGAN leaf images dataset. An average enhancement in the precision score of 51% using the CycleGAN leaf images dataset in the three deep vision classification models.

**Figure 7:**
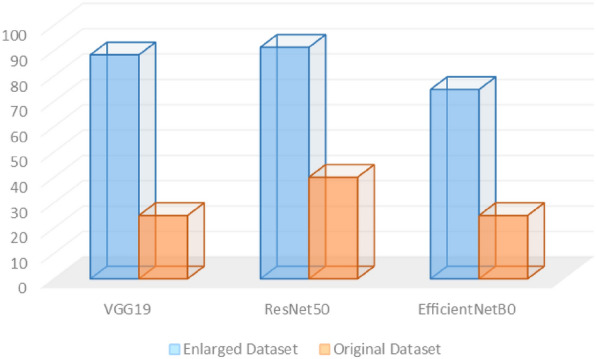
The recall score for the 3 models using the two datasets.

The recall score on the other hand was also measured for the two systems using the three deep vision classification models. The recall score has noticeably enhanced when adopting the CycleGAN leaf images dataset. Figure 7 presents the recall score values for the two systems using the three deep vision classification models. As noticed, an average enhancement of 57% in the recall score value over the recall score that was obtained using the original dataset.

**Figure 8:**
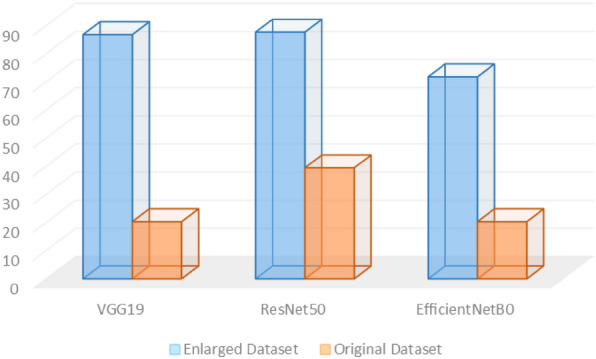
The F1-score for the 3 models using the two datasets.

Finally, the F1-score was measured for the two systems using the three different deep vision classification models. Figure 8 depicts the F1-scores for the three deep vision classification approaches using the original dataset and the CycleGAN leaf images dataset. As noticed the F1-score values have noticeably enhanced when the CycleGAN leaf images dataset was employed. An average enhancement of 48% when employing the CycleGAN leaf images dataset with the three deep vision classification models.

## Discussion

In general, CNN architecture requires a large dataset for training purposes in order to achieve better classification performance, which is not valid in the area of plant disease classification. In addition, when the number of model parameters exceeds the number of data samples, the small training dataset causes overfitting, and hence minimize the classification accuracy^32^.

Data augmentation can overcome the challenges of insufficient data and unequal distribution through increasing the model performance for several tasks, including: detection, classification and recognition of plant diseases. According to^33^, most of the recent developed machine learning approaches are based on the assumption that training sets have a well-balanced distribution, which is not exist in several case studies. Authors of^34^ proved that class imbalance has an impact on feed-forward neural network performance, particularly as the complexity of classes increases.

Moreover, data augmentation has been employed in diverse deep learning applications due to its capability of creating new training data from the existing data. This is commonly achieved through applying annotation-preserving changes to the input data, such as: rotating, deforming, or translation of the image. Data augmentation can generate an infinite amount of training data. One of the efficient data augmentation approaches is the elastic deformation which has been effectively employed with CNN for medical image segmentation^1^.

By overcoming the challenges of insufficient data and unequal distribution, data augmentation can increase model performance for tasks including classification, detection, and recognition of these diseases. Most learning algorithms are based on the assumption that training sets have a well-balanced distribution, which is not necessarily the case. The presented work in this paper has overcome the challenge of insufficient images to be involved in the training process.

VGG19, ResNet50, and EfficientNetB0 have different classification performance when applied CycleGAN on the original data set, therefore the images generated by CycleGAN are quite similar to the original images, while CyleGAN based on random noise can generate various images. Furthermore, because healthy and curl were not even detected in the initial dataset, the overall classification accuracy for healthy and curl was higher than it had been previously. As a result, the experiment demonstrates that utilizing CycleGAN to produce plant leaf images to expand the dataset has practical utility since it can generate a variety of data, thereby resolving the issue of an unbalanced data set, and hence improving the classification accuracy for pear leaf disease classification.

## Conclusion and future work

In the field of image generation, the CycleGAN architecture is commonly employed for diverse types of applications. However, most of the existing CycleGANs approaches were utilized to create images with sufficient data. The goal of this work was to build and employ the CycleGAN architecture for producing pear leaves in order to enlarge the data set and assess the usability of the generated leaves based on classification accuracy. In the selected dataset, the Curl and healthy leaves were unbalanced in the pear disease database. Therefore, we utilized the CycleGAN approach to generate 224 × 224 pixels leaf images through utilizing a minimal data source. After considering several experiments, the CycleGAN architecture has significantly enhanced the classification accuracy for the pear disease classification. In addition, the recall, precision, F1-score metrics have also been improved using the CycleGAN leaf dataset. For future works, we aim to investigate additional deep vision approaches for the purpose of improving the classification accuracy. Moreover, we aim to develop a reliable system using small-size processors (for instance: Raspberry Pi 4 or Jetson Nano) for the purpose of real-time plant disease classification.

### Plant guidelines

The collection of plant material and the performance of experimental research on the pear plants complied with the national guidelines of the Kingdom of Saudi Arabia.

## Data Availability

The dataset that has been used in this study is available in https://zenodo.org/record/5557313.
